# Supplementary material for: The genome of pest Rhynchophorus ferrugineus reveals gene families important at the plant-beetle interface
Source: Commun Biol. 2020 Jun 24;3:323. doi: 10.1038/s42003-020-1060-8 (PMC7314810; doi:10.1038/s42003-020-1060-8)

Evolution of the gene family "3367" (p=0.022)

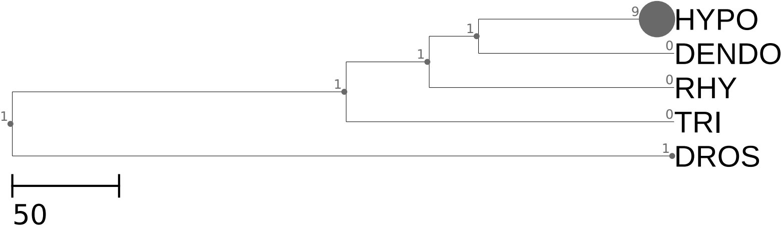

Supplement: Supplementary file 18 — Supplementary data file 13 [file 42003_2020_1060_MOESM18_ESM.zip › Additional_file_12/3367.pdf]
